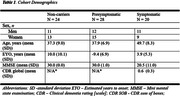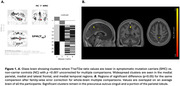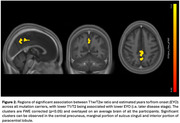# T1/T2 weighted ratio mapping to assess intracortical myelination in autosomal dominant Alzheimer's disease

**DOI:** 10.1002/alz.091752

**Published:** 2025-01-09

**Authors:** Alessia Sgalia, Dilek Ocal, Thomas Veale, Damien Ferguson, Antoinette O'Connor, Ian B. Malone, David M Cash, Nick C Fox, Philip SJ Weston

**Affiliations:** ^1^ University College London, London, London UK; ^2^ Dementia Research Centre, UCL Queen Square Institute of Neurology, London UK; ^3^ Dementia Research Centre, UCL Queen Square Institute of Neurology, London, London UK; ^4^ Dementia Research centre, University College London, London UK; ^5^ Dementia Research Centre, UCL Queen Square Institute of Neurology, University College London, London UK; ^6^ Dementia Research Centre, Department of Neurodegenerative Disease & UK Dementia Research Institute, Institute of Neurology, University College London, London UK

## Abstract

**Background:**

Identifying early pathological changes in Alzheimer’s Disease (AD), and accounting for variability in progression between individuals, is crucial for timely and effective intervention. While Amyloid‐b, tau, and neuronal loss are core pathological processes in AD with established biomarkers, myelin alterations have been shown to occur but remain under‐investigated. The T1/T2w ratio represents an accessible MRI‐based marker, providing a potential proxy measure of myelin integrity. Autosomal dominant AD (ADAD) provides a unique opportunity to investigate AD‐related changes across its continuum, estimating time to/from symptom onset from family history. This study examines the T1/T2‐weighted ratio as an indicator of extra‐neuronal AD pathology in ADAD.

**Method:**

Seventy‐two ADAD family members were included: 28 presymptomatic mutation carriers, 20 symptomatic mutation carriers and 24 non‐carrier controls (Table 1). Volumetric T1 and T2‐weighted MRI sequences were acquired. Whole brain preprocessing and T1/T2w ratio were calculated for each participant using MRtool in SPM12 (bias correction, rigid registration, calibration, ratio), followed by tissue segmentation, DARTEL, normalisation and smoothing. Voxel‐wise analysis compared T1/T2 across 1) presymptomatic mutation carriers vs. non‐carriers and 2) symptomatic mutation carriers vs. non‐carriers, adjusting for age and sex. In mutation carriers only, we assessed correlations between T1w/T2w ratio and 1) estimated years to/from onset (EYO), and 2) Mini‐Mental State Examination (MMSE) scores.

**Result:**

Symptomatic ADAD mutation carriers had lower T1w/T2w ratios than non‐carriers in the medial parietal lobe (t = 7.06, p <0.001 family‐wise error (FWE) corrected) (Figure 1), likely reflecting altered myelin integrity. After FWE correction, no significant differences were observed between presymptomatic mutation carriers and non‐carriers. Across all mutation carriers, there was a positive correlation between T1w/T2w and EYO (i.e. lower T1/T2 was associated with less time to symptom onset), with significant clusters in the praecuneus (p < 0.05 FWE‐corrected) (Figure 2). There was a positive correlation between T1w/T2w and MMSE (t= 5.82, p <0.05 FWE‐corrected) (Figure 3).

**Conclusion:**

T1w/T2w ratio is reduced in ADAD, suggesting reduced myelin integrity, and is associated with measures of disease stage (EYO) and severity (MMSE). T1w/T2w ratio could therefore provide a feasible biomarker of myelin integrity, potentially enhancing early AD prognostication, monitoring and treatment development.